# Human Biomonitoring Initiative (HBM4EU): Human Biomonitoring Guidance Values Derived for Dimethylformamide

**DOI:** 10.3390/toxics10060298

**Published:** 2022-05-31

**Authors:** Farida Lamkarkach, Matthieu Meslin, Marike Kolossa-Gehring, Petra Apel, Robert Garnier

**Affiliations:** 1 ANSES 14 Rue Pierre et Marie Curie, 94701 Maisons-Alfort, France; matthieu.meslin@anses.fr; 2 German Environment Agency (UBA), Corrensplatz 1, 14195 Berlin, Germany; marike.kolossa@uba.de (M.K.-G.); petra.apel@uba.de (P.A.); 3 Paris Poison Centre, Toxicology Department (FeTox), APHP, Lariboisière-Fernand-Widal Hospital, 200 Rue du Faubourg Saint-Denis, 75010 Paris, France; robert.garnier@aphp.fr

**Keywords:** HBM4EU, dimethylformamide, DMF, HBM-GV, guidance value, biomarker, human biomonitoring, toxicokinetics, health effects, liver, carcinogenicity, reprotoxic effects

## Abstract

Within the European Joint Program on Human Biomonitoring HBM4EU, human biomonitoring guidance values (HBM-GVs) for the general population (HBM-GV_GenPop_) or for occupationally exposed adults (HBM-GV_Worker_) are derived for prioritized substances including dimethylformamide (DMF). The methodology to derive these values that was agreed upon within the HBM4EU project was applied. A large database on DMF exposure from studies conducted at workplaces provided dose–response relationships between biomarker concentrations and health effects. The hepatotoxicity of DMF has been identified as having the most sensitive effect, with increased liver enzyme concentrations serving as biomarkers of the effect. Out of the available biomarkers of DMF exposure studied in this paper, the following were selected to derive HBM-GV_Worker_: total N-methylformamide (tNMF) (sum of N-hydroxymethyl-N-methylformamide and NMF) and N-acetyl-S-(N-methylcarbamoyl)cysteine (AMCC) in urine. The proposed HBM-GV_Worker_ is 10 mg·L^−1^ or 10 mg·g^−1^ creatinine for both biomarkers. Due to their different half-lives, tNMF (representative of the exposure of the day) and AMCC (representative of the preceding days’ exposure) are complementary for the biological monitoring of workers exposed to DMF. The levels of confidence for these HBM-GV_Worker_ are set to “high” for tNMF and “medium-low” for AMCC. Therefore, further investigations are required for the consolidation of the health-based HBM-GV for AMCC in urine.

## 1. Introduction

The European Joint Program on Human Biomonitoring (HBM4EU) is a joint effort of 30 countries and the European Environment Agency, co-funded by the European Commission, within the framework of Horizon 2020 [1]. With a project duration from 2017 to 2022, HBM4EU aims to harmonize and advance human biomonitoring in Europe by studying the internal exposure of European citizens to chemicals and its impact on health according to jointly agreed-upon harmonized procedures. The project generates scientific knowledge to answer concrete policy-relevant questions for Europe and thus builds bridges between science and policy, benefiting society by improving public health.

HBM-GVs are derived according to the methodology set within HBM4EU and detailed in Apel et al. (2020). Values are specifically derived for the general population (HBM-GV_GenPop_) and for workers (HBM-GV_Worker_). They indicate the concentration of a compound or its metabolite(s) in a biological matrix (e.g., blood, urine) at and below which no health risk is anticipated (according to current knowledge) [2].

The HBM4EU Consortium identified eighteen substances or substance groups of high priority to answer open policy-relevant questions via targeted research. HBM-GVs have previously been established for several substances (e.g., cadmium [3], BPA [4], phthalates and DINCH [5], and pyrrolidones [6]). The selected substances included DMF as part of the aprotic solvent group.

As DMF is a widely used chemical in numerous industrial sectors, many workers are expected to be occupationally exposed to it. DMF is readily biodegradable and not known for its bioaccumulation potential; thus, long-term environmental exposure is unlikely [7]. Therefore, HBM-GVs are proposed only for workers (HBM-GV_Worker_) in the present work.

DMF is predominantly used as an industrial solvent in the synthesis of fine chemicals (e.g., active pharmaceutical ingredients and crop protection ingredients) and for the production of polyurethane-coated textiles (e.g., artificial leather, rain protection clothing, footwear, medical mattress covers, surgical incise films, etc.). DMF is also used as a solvent in the production of synthetic fibers and for the formulation of mixtures, as a gas stabilizer in acetone cylinders, as a cleaning solvent, as a laboratory chemical, etc. [8]. In 2019, the Risk Assessment Committee (RAC) and the Committee for Socio-economic Analysis (SEAC) of ECHA emitted an opinion on an Annex XV dossier proposing restrictions on DMF [7]. RAC concluded that “manufacturers, importers and downstream users of the substance on its own (regardless of whether DMF is a (main) constituent, an impurity or a stabilizer) or in mixtures in a concentration equal or greater than 0.3% shall use in their chemical safety assessment and safety data sheets a worker based harmonized Derived No Effect Level (DNEL) value for long-term inhalation exposure of 6 mg·m^−3^ and a worker based harmonized DNEL for long-term dermal exposure of 1.1 mg·kg^−1^ bw·d^−1^.” [7].

According to the harmonized classification, labelling, and packaging (CLP) regulation (EC N° 1272/2008), DMF may damage an unborn child, is harmful in contact with skin, can cause serious eye irritation, and is harmful if inhaled. Until 2018, DMF was not classified as to its carcinogenicity to humans by the International Agency for Research on Cancer (IARC) due to inadequate evidence in humans and evidence suggesting lack of carcinogenicity in animal studies [9]. In 2018, IARC re-evaluated DMF as probably carcinogenic for humans (Group 2A) based on limited evidence in humans and sufficient evidence in experimental animal studies [10]. DMF is not categorized for its carcinogenic properties in the European Union.

For the present work, a review of health effects associated with DMF exposure was conducted to provide an HBM-GV_Worker_ for occupationally exposed people, with respect to the current methodology set in the HBM4EU project [2]. To achieve this, an assessment of the extensive database on DMF (including toxicokinetic and toxicodynamic data) was performed.

## 2. Materials and Methods

### 2.1. General Methodology to Derive HBM-GVs in the Framework of the HBM4EU Project

An HBM-GV corresponds to a biomarker concentration in a biological matrix and represents a value at and below which adverse human health effects generated by the substance exposure are not to be expected, according to current knowledge. HBM-GVs are derived within the HBM4EU project according to a systematic and transparent methodology [2] and by taking into account the feedback provided by competent experts from the 30 HBM4EU participating countries, which was thereby mutually agreed upon within the HBM4EU consortium.

The methodological approach was developed on the basis of the procedure described in the German Human Biomonitoring Commission’s position paper [11,12], by the team from the Summit Toxicology consulting firm [13,14], and in the guidance document elaborated by the French Agency for Food, Environmental, and Occupational Health and Safety (ANSES) [15].

**Figure 1:**
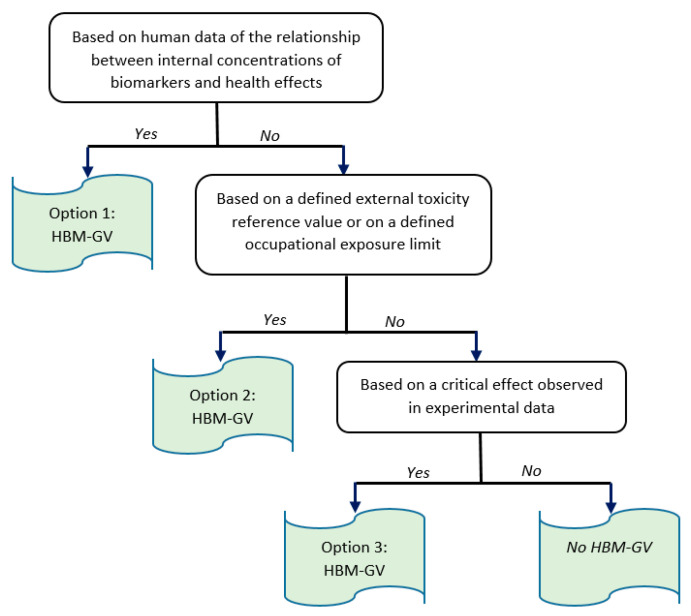
Decision tree for determining HBM-GVs.

The HBM4EU method for deriving an HBM-GV can be divided into the following steps:

- Selection of the relevant biomarker(s): a biomarker is defined as any substance, structure, or process that can be measured in the body or its degradation product(s) which influences or predicts the incidence of outcome or disease. Biomarkers can be classified into biomarkers of exposure (BME), biomarkers of effects, or biomarkers of susceptibility [2]. This first step consists of the data collection on the substance and its metabolites (i.e., toxicokinetic and toxicodynamic data). Based on these data, biomarkers of exposure and/or effect are identified and then chosen according to defined criteria: specificity, sensitivity, half-life, sampling conditions, invasiveness, background level, and analytical methods [15].
- The derivation of HBM-GVs for the selected biomarkers can then be conducted through three possible options (decision tree described in Figure 1). When the corresponding data are available, the preferred option is to base HBM-GV(s) identification on the relationship between internal concentrations of the selected biomarker(s) and the occurrence of adverse effects. The second possible option is to derive HBM-GVs from external limit values (i.e., Occupational Exposure Levels [OEL] or Toxicity Reference Values [TRV]) proposed by relevant European or non-European bodies. The last option consists of the derivation of HBM-GVs on the basis of critical effects observed in animal toxicological studies. These options are described in more detail in Apel et al. (2020) [2].

If possible, option 1 is preferred for deriving an HBM-GV (Figure 1). For this, the following steps are required:

- Choice of the critical effect which is considered to be the most sensitive among all adverse effects that may arise from exposure to the substance (e.g., changes in morphology, physiology, growth, development, reproduction, or life span resulting in an impairment of functional capacity, an impairment of the capacity to offset additional stress, or an increase in sensitivity).
- Selection of the key study and identification of a point of departure (POD) with the most informative studies, i.e., well-conducted human studies adequately reporting measured internal concentration levels of a substance, sampling times, analytical methods used, and the relationships between concentrations of a substance or its metabolites in human biological media and the occurrence of adverse effects. If relevant and qualitatively acceptable human studies are available, a key human study together with a Point of Departure (POD) is selected.
- Application of assessment factors (AFs), when necessary, to obtain the HBM-GVs. These can be divided into an AF_H_ for the intraspecies variability or possible other AFs to compensate for the potential remaining uncertainties in the derived HBM-GV, especially regarding the possible deficiencies or data gaps in the available data sets [2].

### 2.2. Methodology Used for Deriving HBM-GV_Worker_ for DMF

For the present work, the general methodology to derive HBM-GVs in the framework of the HBM4EU project was applied for DMF using the data issued from:

- the reports by the American Conference of Governmental Industrial Hygienists (ACGIH) [16,17], the German Research Foundation or Deutsche Forschungsgemeinschaft (DFG) [18,19], the European Chemicals Agency (ECHA) [7], the International Agency for Research on Cancer (IARC) ([10]), and the Scientific committee for occupational exposure limits (SCOEL) [20];
- for more recent and specific publications, a bibliographical research, which was conducted in Medline and Scopus until 2021 with the following keywords: Dimethylformamide, DMF, guidance value, toxicity reference value (TRV), biomarker of exposure, biomonitoring, toxicokinetic, health effects, liver, carcinogenicity, and reprotoxic effects.

Additionally, a global level of confidence (i.e., high, medium, or low) is attributed to each derived HBM-GV_Worker_ to reflect the uncertainties related to its derivation. This level of confidence is mainly based on the quality of the available data [2].

## 3. Results

### 3.1. Identification of Possible Biomarkers of Exposure

DMF absorption has been well-studied in humans (workers and volunteers); absorption via inhalation is high, with 60–90% of the inhaled dose retained in the respiratory tract [21,22]. Dermal absorption in humans from direct contact is also very high (up to 40%, depending on temperature and humidity) [23,24,25]. In the study by Nomiyama et al. (2001), in which the authors evaluated the difference between the absorption of DMF vapor by dermal and inhalation routes, it was estimated that skin and lung absorption contributed for 40.4% and 59.6% of total absorption, respectively [26]. In a study conducted in exposed workers, Lauwerys et al. (1980) reported that in workers without gloves, the amount of DMF absorbed through the skin may be more than twice that absorbed by inhalation [27].

DMF and its metabolites are distributed throughout the organism, and quite uniformly in the different tissues [7,16]. In rodents, they freely cross the placenta [28,29]; the corresponding data are not available for humans.

**Figure 2:**
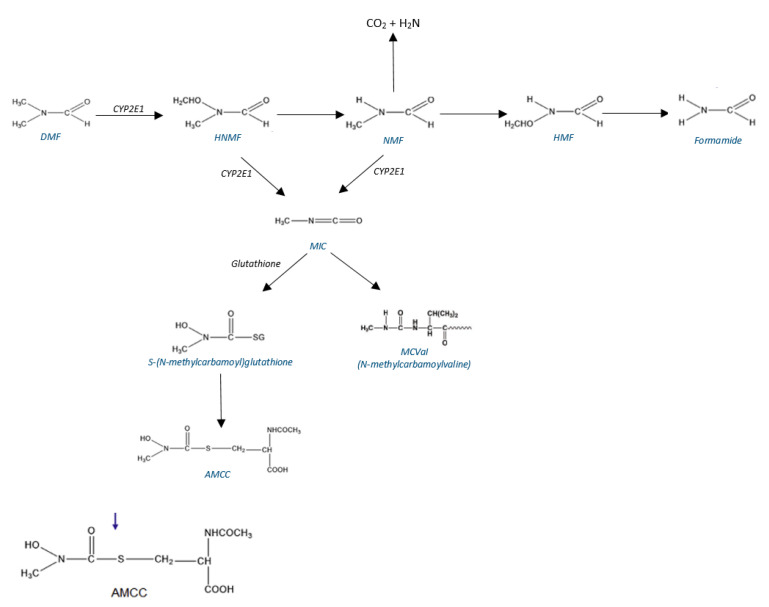
A scheme for the metabolic pathways of DMF (as described in the text).

DMF is rapidly metabolized in the liver. N-hydroxymethyl-N-methylformamide (HMMF) arises from DMF mainly through enzymatic oxidation by the cytochrome P450 enzyme system (CYP2E1) [30]. Then, demethylation leads to the formation of N-methylformamide (NMF). The concentrations of HMMF and NMF in urine are grouped as total NMF (tNMF), as it is difficult to analyze these metabolites separatly due to the thermal decomposition of the hydroxyl derivative in the injection port of the gas chromatograph [31,32]. NMF can be further oxidized to N-(hydroxymethyl)formamide (HMF) and formamide. N-acetyl-S-(N-methylcarbamoyl)cysteine (AMCC) is another metabolite formed after exposure to DMF [33]. AMCC is the end product of the enzymatic breakdown of S-(N-methylcarbamoyl)glutathione. The latter is formed by the reaction between glutathione and the probable reactive metabolic intermediate, methyl isocyanate (MIC) [22,33]. Unlike in rodents, AMCC is a major metabolite of DMF in humans [34] (Figure 2).

As MIC is a reactive substance, it also produces adducts to proteins; N-methylcarbamoylvaline (MCVal) adducts at the N-terminal position of the globin chains of hemoglobin are also identified as possible biomarkers of DMF exposure [35,36]. Nε-(N-methylcarbamoyl) lysine adducts to globin were also identified in humans occupationally exposed to DMF [37]; however, data on this possible biological indicator of exposure are very scarce. 

DMF metabolism has been reported to be saturable; it is able to inhibit its own metabolism [7,38,39,40]. It is competitively inhibited by simultaneous alcohol exposure (and potentially by the exposure to any other potential substrate of CYP2E1) [41]. DMF reciprocally inhibits ethanol metabolism; it is a strong inhibitor of aldehyde-dehydrogenase, and this effect is responsible for alcohol intolerance reactions (resulting from the accumulation of acetaldehyde) [42]. Usual alcohol consumption induces CYP2E1 activity and consequently DMF metabolism into HMMF (and probably also into MIC) [7].

The hepatic biotransformation of DMF and the urinary excretion of its metabolites (HMMF, NMF, formamide, and AMCC) are the main elimination pathways of DMF. In a study by Mraz and Nohova (1992), the authors analyzed DMF metabolite excretion in 10 volunteers during and after an 8-h exposure to DMF. After exposure to 30 mg·m^−3^ or 60 mg·m^−3^, the yield of compounds measured in urine was similar: 0.3% DMF, 22.3% tNMF (HMMF + NMF), 13.2% tHMF (HMF + formamide), and 13.4% AMCC. The half-lives of tNMF, AMCC, tHMF, and DMF were 4, 23, 7 and 2 h, respectively, after 30 mg·m^−3^ exposure [22]. A slower elimination, without significant accumulation over the workweek, was reported after skin exposure of volunteers to liquid DMF with an average half-life of 7–8 h for tNMF [23,24].

Saillenfait et al. (1997), who treated lactating rats with a single oral dose of 100 mg/kg bw [^14^C]-DMF on lactation day 14, measured DMF, HMMF, and NMF in the rats’ milk at concentrations equal to those in the plasma. In addition, they noted that 60–70% of the absorbed dose of [^14^C]-DMF was excreted in the urine and 3–4% in the feces [29].

There is no study evaluating the pulmonary elimination, possibly due to strong adsorption of DMF on the walls of the measuring device [22].

A recent overview of key studies on toxicokinetic data in humans and animals is presented in ECHA’s opinion on DMF restriction [7].

Based on all these data, several metabolites can be identified as potential BME for biological monitoring of DMF at the workplace:

- Unchanged DMF in urine;
- total NMF or tNMF (which is the sum of HMMF and NMF) in urine;
- AMCC in urine;
- MCVal in blood; and
- formamide in urine.

Advantages and limits of each BME are detailed in Section 3.4.

### 3.2. Identification and Characterization of the Dangers Associated with DMF Exposure

As the HBM4EU strategy for deriving an HBM-GV recommends giving priority to human data for the characterization of the relationship between the risk of adverse effects and internal concentrations of the selected biomarker(s), only human data were initially considered. This initial retrieval provided a sufficiently extensive database on the effects on humans, allowing the identification of HBM-GVs.

In published human studies of health effects, DMF exposure was mostly assessed via airborne DMF measurements and/or via biomonitoring (mainly tNMF and AMCC in urine, and less often MCVal in blood).

The following effects have been studied: acute toxicity; skin, eye, and respiratory tract irritation; chronic toxicity (effects on liver, gastro-intestinal effects, and alcohol intolerance); genotoxicity; carcinogenicity; and reproductive and developmental effects.

A few publications have reported on DMF irritative effects at the workplace; effects such as chemical burns of the skin and eyes were observed after direct contact with liquid DMF [43]. Workers reported eye and upper airway irritation after exposure to 10 ppm or more [17].

It appears that the main systemic target for toxicity after acute or chronic exposure to DMF is the liver, both in humans and laboratory animals. Experimentally, the NOAEL and LOAEL for hepatotoxicity in the most sensitive species are 12 mg·kg^−1^ bw·d^−1^ and 60 mg·kg^−1^ bw·d^−1^, respectively, for the ingestion route, and 25 and 100 ppm, respectively, for the inhalation route, the rat being the most sensitive species in both cases [18]. In humans, acute high-dose exposure to DMF can cause damage to the liver and even lead to death, although in most reported cases, the damage was reversible [44]. He et al. (2010) observed that DMF could produce acute toxic hepatitis as well as chronic hepatic damage such as hepatic cirrhosis [45]. More recently, Wu et al. (2017) reported that liver damages in DMF-exposed workers include hepatitis, liver fibrosis, and cirrhosis [46]. Many authors investigated liver function via serum liver enzyme concentrations (e.g., alanine aminotransferase (ALT); aspartate aminotransferase (AST); and gamma glutamyltranspeptidase (γGT)) together with subjective symptoms and clinical signs in workers exposed to DMF. According to the MAK commission, an increase of serum AST and ALT is the most suitable parameter for detecting DMF-induced effects on the liver [18].

Table 1 presents those studies where biological evaluation of exposure and a search for hepatic damage was performed. In some of them, DMF exposure was also evaluated through air measurements. However, DMF airborne concentrations are not always consistently associated with the occurrence of health effects, as DMF systemic contamination may also result from direct skin contact and vapor–skin absorption [45].

Some authors considered confounding factors, e.g., alcohol consumption (based on questionnaires, in almost all cases). Workers who did not consume alcohol tolerated much higher concentrations of DMF without changes in liver functions [47,48].

Several studies have reported DMF-related disorders of the digestive system; the symptoms included abdominal pain, anorexia, nausea, vomiting, and diarrhea [28]. In some studies, these effects on the digestive tract were reported in workers exposed to low DMF air concentrations (below 10 ppm or even 5 ppm), but with probable direct skin contact [49,50,51].

**Table 1:** Summary of occupational studies reporting health effects of DMF exposure with biomonitoring data.

Reference	Subjects	Exposure DMF in the Air (DMFa) Metabolites *		Results/Observations
Lyle et al., 1979 [52] England	Workers (DMF used as solvent) N = 102) 3-year follow-up	DMFa Range: <10 to 200 ppm (30–600 mg·m^−3^) tNMF (tNMFu) Range: <10 to 77 µL/L *(probable error in the unit)*		Alcohol intolerance reactions Facial flushing and other symptoms in 19 workers 26 of the 34 episodes occurred after the workers had consumed alcoholic drinks Liver function not investigated
Yonemoto and Suzuki, 1980 [53] Japan	Workers (synthetic leather factory) N = 11 (biomonitoring data for 9 of them)	DMFa Range: 0–5 ppm (0–15 mg·m^−3^) (TWA) Post-Shift (PS) tNMFu Range: 0.4–19.56 mg·d^−1^		No effect on serum biochemistry (liver enzymes) Alcohol intolerance: 6/11 workers said to be less tolerant than before
Lauwerys et al., 1980 [27] Belgium	Workers in an acrylic fiber factory N = 22 (+28 controls)	DMFa Mean: 13 (1.3–46.6) mg·m^−3^ (4.5 (0.4–15.3) ppm) Stationary sampling tNMFu <40–50 mg·g^−1^ cr (PS)		No effects on serum biochemistry (liver enzymes not elevated) Signs of alcohol intolerance in some workers
Catenacci et al., 1984 [54] Italy Quoted by SCOEL (2006) [20]	N = 54 (employed > 5 y) acrylic fiber plant 2 groups exposed and 54 controls)	Group 1 (N = 28) DMFa Mean (range): 6 (4–8) ppm (18 (12–24) mg·m^−3^) tNMFu 22.3 mg·L^−1^	Group 2 (N = 26) DMFa Mean (range): 1 (0.6–1.6) ppm (3 (1.8–4.8) mg·m^−3^) tNMFu: 7 mg·L^−1^	No significant effects on liver enzymes in the 2 groups
Sakai et al., 1995 [55] Japan	Workers (N = 10) Polyurethane production 2.5-year follow-up	DMFa Geometric mean (GM): 2.5–10.4 ppm (7.5–31.2 mg·m^−3^) PS tNMFu Mean: 24.7 mg·g^−1^ cr AMCCu Mean: 22.0 mg·g^−1^ cr		No effects on liver enzymes
Fiorito et al., 1997 [50] Italy	N = 75 (employed) synthetic leather production and 75 controls (unexposed workers)	DMFa Group 1 (Washing) N = 10 GM: 21.5 mg·m^−3^ (7.2 ppm) Range: 5–40 mg·m^−3^ Group 2 (Production) N = 12 GM: 18.7 mg·m^−3^ (6.2 ppm) Range: 5–40 mg·m^−3^ tNMFu (N = 22): GM: 13.6 mg·L^−1^ or 13.4 mg·g^−1^ cr PS		Elevation of liver enzymes (12/75) [*p* < 0.01] Alcohol intolerance in 50% of exposed workers and facial flushing (38%), palpitations (30%), headache (22%), body flushing (15%), and tremors (14%) Gastrointestinal symptoms (stomach pain, nausea, loss of appetite) in 50% of exposed workers
Wrbitzky and Angerer, (1998) [47]; Wrbitzky, (1999) [48] Germany	Polyacrylic fiber production N = 126 (total of exposed workers)	DMFa Mean (SD): 4.1 ± 7.4 (<0.1–37.9) ppm (12.3 ± 22.2 mg·m^−3^) tNMFu Mean (SD): 14.9 ± 18.7 (0.9–100) mg·L^−1^ 9.1 ± 11.4 (0.5–62.3) mg·g^−1^ cr		Effects on liver enzymes Synergetic effect of alcohol consumption on liver enzymes activity
	Finishing N = 55	DMFa Mean (SD): 14.2 ± 2.2 (>0.1–13.7) ppm (42.6 ± 6.6 mg·m^−3^) tNMFu Mean (SD): 4.5 ± 4.3 mg·g^−1^ cr		Effects on liver enzymes in alcohol consumers
	Dyeing N = 12	DMFa Mean (SD): 2.5 ± 3.1 (0.1–9.8) ppm (7.5 ± 9.3 mg·m^−3^) tNMFu Mean (SD): 6.7 ± 5.4 (0.8–17.2) mg·g^−1^ cr		No effects on liver enzymes in workers not drinking alcohol Reduced alcohol consumption in workers drinking alcohol
	Dry spinning N = 28	DMFa Mean (SD): 6.4–9.6 (0.8–36.9) ppm (19.2 ± 28.8 mg·m^−3^) tNMFu Mean (SD): 11.6 ± 13.1 (0.9–62.3 mg·g^−1^ cr)		
	Wet spinning N = 30	DMFa Mean (SD): 7.3 ± 10.2 (0.3–37.9) ppm (21.9 ± 30.6 mg·m^−3^) tNMFu Mean (SD): 16.0 ± 15.9 (0.4–54.0) mg·g^−1^ cr		
He et al., 2010 [45] China	Synthetic leather and other resins production N = 79 (58 men and 21 women)	Group 1 (N = 33): Low exposure DMFa Min-Max: Not detected- <4.55 mg·m^−3^ (1.6 ppm) DMFu GM: 0.26 mg·g^−1^ creatinine tNMFu GM: 1.80 mg·g^−1^ creatinine AMCCu GM: 4.25 mg·g^−1^ creatinine Group 2 (N = 24): Medium exposure DMFa (Mean): 9 mg·m^−3^ (3 ppm) DMFu (GM): 0.53 mg·g^−1^ creatinine tNMFu (GM): 9.6 mg·g^−1^ creatinine AMCCu (GM): 25.4 mg·g^−1^ creatinine Group 3 (N = 22): High exposure DMFa (Mean): 36 mg·m^−3^ (12 ppm) DMFu (GM): 1.78 mg·g^−1^ creatinine tNMFu (GM): 26.5 mg·g^−1^ creatinine AMCCu (GM): 45.5 mg·g^−1^ creatinine		About 60% of subjects with urine AMCC concentration above 40 mg·g^−1^ cr had raised liver enzyme activities Statistically more workers with raised liver enzymes in group 3 (high exposure group) than in group 1 (administrative staff of the factory); *p* < 0.05
Kilo et al., 2016 [51] Germany	Synthetic fiber production N = 220 workers and 175 Controls	Mean ± SD DMFa: 6.2 ± 7.6 mg·m^−3^; 2.1 ± 2.5 ppm tNMFu: 7.75 (±8.82) mg·L^−1^ AMCCu: 9.42 (±10.42) mg·g^−1^ cr McVal: 83.3 (±83.1) nmol·g^−1^ globin		None of the tested liver enzyme activities showed a positive association with any of the three exposure markers Alcohol intolerance reactions (not influencing alcohol consumption behavior)
Wu et al., 2017 [46] China	Synthetic leather production N = 698 And 188 controls	3 exposure groups: Median (range) Low exposure group tNMFu (N = 228): 0.0025 mg·L^−1^ (ND-0.11) AMCCu (N = 227): 2.18 mg·L^−1^ (ND-16,95) MCVal (N = 232): 15.19 nmol·mol^−1^ globin (ND-29.37) Moderate exposure groups tNMFu (N = 227): 1.78 mg·L^−1^ (0.11–3.88) AMCCu (N = 228): 44.9 mg·L^−1^ (16.95–86.62) MCVal (N = 234): 46.00 (29.37–63.95) nmol·mol^−1^ globin High exposure groups tNMFu (N = 227): 9.59 mg·L^−1^ (>3.88) AMCCu (N = 227): 148.01 mg·L^−1^ (>86.62) MCVal (N = 232): 87.01 (63.95–) nmol/mol globin		Liver injury assessed by measurement of liver enzyme levels and compared to reference value ranges (AST and ALT: 0–45, γGT: 8–58U/L) Statistically more workers with raised liver enzymes only in high-exposure group for tNMF, in both moderate- and high-exposure groups for AMCCu and MCVal (*p* < 0.05)

* DMFu: DMF in urine; tNMFu: Total NMF in urine; AMCCu: AMCC in urine; MCVal: MCVal in blood; SD: Standard deviation.

Exposure to DMF in combination with subsequent alcohol consumption can induce an alcohol intolerance reaction (with flushing of the facial skin, neck, and arms). This alcohol intolerance results from the accumulation of acetaldehyde due to the inhibition of aldehyde dehydrogenase [18,52]. Alcohol intolerance has been observed after DMF exposure in both rats and humans [51,52,56]. Kilo et al. (2017) observed signs of alcohol intolerance (flushing reaction) in almost half of the workers at 4.43 mg·L^−1^, 4.84 mg·L^−1^, and 60.5 nmol·g^−1^ globin for urine tNMF, urine AMCC, and MCVal adducts to hemoglobin, respectively. Alcohol intolerance can be observed for very low DMF exposure, but with a large interindividual variability due to the corresponding interindividual variability of aldehyde dehydrogenase activity [51]. Wolff (1972) reported that 83% of East Asian subjects (Japanese, Taiwanese, and Koreans) responded with a marked visible facial flushing after drinking small amounts of alcohol. In contrast, after similar doses, only 3% of Caucasian subjects showed visible flushing [57]. Thus, Antabuse effects reported in occupational studies must be interpreted in light of the differences in alcohol sensitivity (based on genetic polymorphisms of the enzymes alcohol dehydrogenase and aldehyde dehydrogenase) [58].

IARC (2018) [10] reviewed a large body of data to assess the genotoxicity of DMF [9,59,60,61,62,63,64,65,66]. Overall, there is no proof of the genotoxicity of DMF; experimental studies both in vitro, in prokaryotes and mammalian cells, and in vivo, in rodents, generally gave negative results. The genotoxic effects reported in a few field studies cannot constitute evidence of genotoxic effects of DMF due to confounding factors, in particular from co-exposures.

According to IARC [10], there is sufficient evidence for the carcinogenicity of DMF in laboratory animals. The carcinogenicity of DMF has been demonstrated in two inhalation (whole body) studies in rats and mice and in one inhalation (whole body) and ingestion (in drinking water) study in male rats. In an inhalation study in mice by Senoh et al. (2004), exposure to DMF increased the incidence of hepatocellular adenoma, hepatocellular carcinoma, and the aggregate of hepatocellular adenoma and carcinoma and hepatoblastoma; this was observed both in male and female mice with a dose relationship effect [67]. A second study, conducted in mice by Malley et al. (1994), provided negative results [68]. A dose-dependent increase in the incidence of hepatocellular adenomas, hepatocellular carcinomas, and aggregated adenoma and hepatocellular carcinomas was observed in rats of both sexes in an inhalation study [67]. The second study conducted by inhalation in rats was negative [68]. In the combined respiratory and oral study by Ohbayashi et al. (2009) in male rats, exposure to DMF increased the incidence of hepatocellular adenomas and aggregated hepatocellular adenoma and carcinoma. An increased incidence of hepatocellular carcinoma was observed in the group treated orally only [69].

The IARC (2018) determined that there is limited evidence of DMF carcinogenicity in humans based on three publications. The first reported a cluster of 3 cases of testicular cancer in 153 mechanics in a US military aviation repair shop [70]. The second study was motivated by the first, and also reported a cluster of three cases of testicular cancer in workers exposed to DMF in a tannery [71]. The third study was a retrospective study conducted in an acrylic fiber factory; it did not identify an increased risk of testicular cancer associated with DMF exposure. However, it showed an excess risk of oropharyngeal cancers [72]. This retrospective study was followed by a case-control study conducted in this factory and three other similar plants. The latter study identified 11 cases of testicular cancer, but did not show an excess risk of this cancer associated with exposure to DMF (OR: 0.99; 95% CI: 0.22–4.44) [73]. More recently and after the last IARC evaluation, the results of a Korean cohort study were published. The cohort was constituted by 11,953 workers with one or more urine tNMF measurements between 2001 and 2004 for DMF occupational exposure biomonitoring. Their mortality was matched with the mortality data of the Korean National Statistical Office and followed up for cancer mortality between 2000 and 2011. DMF exposure was estimated as low, medium, or high, according to tNMF concentrations in urine measuring <7.5 mg·L^−1^, 7.5–15 mg·L^−1^, or >15 mg·L^−1^, respectively. Overall cancer mortality was significantly elevated in medium and high exposure groups with adjusted hazard ratios (HRadj) of 2.72 (95% CI: 1.09–6.81) and ≥2.41 (95% CI 1.03–5.66), respectively. HRadj were also significantly elevated for lung cancer in the medium exposure group (HRadj 14.36, 95% CI 1.41–146.86) and for hepatocellular carcinoma in the high exposure group (HRadj 3.73, 95% CI 1.05–13.24). As exposure evaluation was only transversal and during a brief period, as the cumulated duration of exposure of each worker is unknown, and as major confounding factors including alcohol consumption and smoking status were not taken into account, these results should be interpreted with caution [74].

The data considered by the experts of IARC as constituting limited evidence of the carcinogenicity of DMF in humans and of an increased risk of testicular cancer associated with exposure to this substance would justify more cautious conclusions. Indeed, this evaluation is based on only two clusters of cases, with a negative case-control study. Moreover, the neoplastic lesions observed in the animal studies were localized only in the liver and not in the testis, and could result from DMF hepatotoxicity and capacity for inducing oxidative stress [10]. In addition, there is no evidence that DMF is genotoxic.

DMF is recognized as toxic for reproduction and classified as such according to the CLP regulation. In humans, the study by Chang et al. (2004) on 12 workers in a synthetic leather factory exposed to DMF showed that these workers had reduced sperm mobility when compared to 8 controls. This decrease was proportional to urinary tNMF concentration but not to DMF air concentration. However, considering the small size of the group and the fact that the number of subjects exposed to DMF and having consumed alcohol (8/12; 66.7%) was significantly higher than that of the controls (3/8; 37.5%), these results should be considered with caution [75].

In animals, numerous studies in rodents have demonstrated the effects of DMF on female fertility and on development. In most studies, embryo/foetotoxic effects included a reduction in the body weight of the offspring and a reduction in number and size of litters. Teratogenic effects included various skeletal malformations (especially craniofacial and sternal malformations). In rats, embryo/foetotoxicity generally occurred at maternally toxic doses or concentrations, and teratogenicity was also not reported in the absence of maternal toxicity. However, in mice and rabbits, embryo/foetotoxicity and/or signs of teratogenicity were observed at doses which did not generate maternal toxicity. Exposure to DMF is consistently reported to result in umbilical hernia in rabbit developmental toxicity studies, whereas gallbladder agenesis and sternal malformations were only observed in the two most reliable studies (after dermal and inhalation exposure). The lowest concentration level causing malformations in rabbits was 150 ppm (NOAEC: 50 ppm) [76].

Based on these results, the RAC concluded that DMF was responsible for skeletal developmental disorders in all three species (rats, mice and rabbits), the rabbit being the most sensitive species to developmental toxicity of DMF [7]. In oral studies the NOAEL for effects on fertility was 219 mg/kg bw/d in mice [77] and the NOAEL for effects on fertility and development was 166 mg/kg bw/d in rats [76]. In respiratory toxicity studies, the LOAEC for developmental effects was 150 ppm in rabbits, with a 50 ppm NOAEC [7,76]. In dermal exposure studies, it was not possible to identify a NOAEL, the LOAEL was 94 mg/kg bw/d in rats [76]. According to the RAC, the transposability to humans of the effects observed in animals is plausible [7]. Orally, the NOAEL in rabbits was 44.1 mg·kg^−1^ bw/d^−1^.

### 3.3. Choice of the Critical Effect

In exposed workers, the critical systemic effects of DMF (i.e., those occurring for the lowest exposure levels) are the hepatotoxic effects.

The results of studies in animals showed hepatotoxic, reproductive, and carcinogenic effects as the most relevant. Therefore, for these endpoints, lower points of departure found in animals are detailed in Table 2.

DMF is responsible for reprotoxic effects. As shown in Table 2, the LOAELs for these effects in the most sensitive species are slightly higher than the corresponding values for hepatotoxic effects, by inhalation (which is the relevant route of exposure for workers).

DMF produced carcinogenic effects in rats and mice. However, DMF is probably not a genotoxic substance; the tumors induced in laboratory animals were always hepatic and occurred after repeated exposure to hepatotoxic doses. From these observations, it may be inferred that DMF is probably a threshold carcinogen, and as highlighted in Table 2, protection against hepatotoxic effects would also protect against carcinogenic effects.

DMF can induce alcohol intolerance in some individuals at lower levels than those responsible for hepatotoxicity. This is documented by numerous case reports and epidemiological studies. However, the great interindividual variability of alcohol tolerance and the indirect character of this adverse effect (which needs alcohol intake to occur) makes it unsuitable as a critical effect, for the fixation of reference exposure limits applicability to all workers.

Considering that hepatotoxic effects are the critical effects of DMF exposure, hepatic enzyme activity (AST and ALT) is a suitable parameter for detecting DMF toxicity in exposed workers.

### 3.4. Choice of Relevant Biomarkers

The possible biomarkers for DMF exposure surveillance were previously identified (see above Section 3.1). The available data on the associations between these biomarkers and health effects in exposed workers are presented in Table 1.

In this section, advantages and limits of each possible BME are described in order to select one or more reference BME(s) on the basis of their specificity, sensitivity, and adequacy to occupational exposure biomonitoring, or the availability of analytical methods.

#### 3.4.1. Unchanged DMF in Urine

Unchanged DMF can be detected in the urine of occupationally exposed people. It is a specific BME to DMF. However, unchanged DMF is excreted only at low levels; there are very few data on urine DMF in exposed workers or volunteers, and due to its very short half-life, this BME is not adapted to usual occupational exposure.

#### 3.4.2. Total NMF in Urine

The excretion half-life of tNMF in urine is short (4 h). Therefore, the concentration of urinary tNMF at end of the shift represents the exposure during a working day, and urine samples for biomonitoring can be collected at the end of any shift or exposure period. The suitability of this biomarker for the derivation of an HBM-GV_Worker_ has been confirmed by the observed positive association between tNMF concentration in urine and health effects in workers, reported by occupational studies over several decades. These studies provide data on tNMF levels in urine associated with liver effects [27,45,46,48,50,51,54,55]. According to Wu et al. (2017), tNMF is the best biomarker for predicting liver injury, based on the statistical significance of differences between workers exposed to DMF with and without liver injury (*p* = 0.001, 0.054, and 0.043, for tNMF, AMCC in urine, and MCVal in blood, respectively) [46]. A good correlation between tNMF in urine and individual airborne DMF concentration has been reported by some authors [27,32,79,80,81,82]. Urinary tNMF could not be detected using gas chromatography, with a limit of detection of 0.1 mg·L^−1^ in people who were not exposed to DMF according to Will et al. (1997) [83]. That makes urinary tNMF a good and specific biomarker of DMF occupational exposure, especially as non-occupational exposure is rare. However, it should be noted that tNMF elimination may be delayed after skin absorption, as shown by Mràz and Nohovà (1992) [22], and in the case of concomitant alcohol consumption [41]. Moreover, some analytical issues are raised by Kawai et al. (1992), who reported that results can be affected by the temperature of the gas chromatography injection port (gas chromatographic methods are commonly used for the analysis of tNMF in urine samples of exposed workers) [32]. Therefore, to obtain complete degradation of HMMF into NMF, the recommended temperature is 250 °C or above [16].

#### 3.4.3. AMCC in Urine

The excretion half-life of AMCC in urine is long (23 h). This results in the progressive elevation of urinary concentration of AMCC over consecutive days of exposure. Therefore, urine sampling for biomonitoring of occupational DMF exposure should be performed at the end of the shift and at the end of the workweek (or at least after several previous shifts) to reflect the cumulative DMF load of the preceding working days.

AMCC urinary level is associated with health effects, especially with hepatotoxicity; AMCC in urine reflects the production of MIC, the presumed reactive intermediate of DMF. AMCC elimination in urine is not delayed by skin exposure [16]. There are limited data showing that AMCC urinary levels are reduced after alcohol consumption due to the inhibition of DMF metabolism [16]. Occupational studies showed positive associations between levels of urinary AMCC and abnormal liver function [45,46,51,55] and between airborne DMF and urinary AMCC levels [55,79,81,82]. According to He et al. (2010), AMCC is an ideal biomarker of exposure in health risk assessments following exposure to DMF, as it is highly correlated with the production of its hepatotoxic metabolite(s) [45].

However, AMCC can be found in urine at low levels in the general population, as reported by Käfferlein and Angerer, Schettgen et al. (2008), and Kenwood et al. (2021), especially in active or passive smokers, as MIC is present in cigarette smoke [84,85,86].

#### 3.4.4. MCVal Adducts to Globin in Blood

MCVal has emerged as another possible biomarker of exposure and possibly as a biomarker of effect for DMF. Since adducts accumulate over the lifetime of the erythrocyte (120 days), MCVal can reflect cumulated DMF exposure over the preceding 4 months. However, due to its kinetics, MCVal requires approximately 100 days to reach a steady state. It is therefore a long-term exposure indicator, for which sampling should only be conducted after several months of exposure.

Two recent studies reported a positive relationship between MCVal and liver effects [46,51]. Seitz et al. (2019) showed a correlation between levels of MCVal in workers and airborne DMF concentration. As for AMCC, the formation of MCVal is directly linked to the presumed reactive intermediate, MIC [82].

For some authors, despite practical and technical difficulties linked to this BME, MCVal is the best biomarker reflecting both cumulated DMF exposure of the last months and hepatotoxic risk. The stability of MCVal (compared to tNMF and AMCC) is very important in some scenarios for risk assessment (episodic or irregular exposure, residents living around factories, or workers recently losing or leaving their jobs). Hepatic damage may persist several days or weeks after an overexposure, which has become undetectable through urine tNMF or even AMCC measurements. For example, Wu et al. (2017) observed five workers with abnormal liver enzyme activities who had low levels of tNMF but high concentration of MCVal [46].

#### 3.4.5. Formamide in Urine

There are limited data on formamide as a biomarker of DMF exposure. Moreover, urine formamide is not a specific biomarker of DMF exposure, as it may also reflect NMF or formamide exposure as shown by Mràz and Nohovà (1992), who detected formamide in urines of control workers [24].

#### 3.4.6. Conclusion on BME Selection

Table 3 summarizes the advantages and limits of each potential BME.

There are not enough data on the associations between unchanged DMF or formamide in urine and health effects or external exposure to derive HBM-GV_Worker_ for these BMEs.

Despite the theoretical advantages presented by MCVal, the paucity of data on the associations of this potential biomarker with external exposure and health effects, together with the invasive character of the associated sampling and the high technical demands, do not allow the selection of this BME for deriving HBM-GVs.

Among the possible BMEs of DMF exposure, total NMF and AMCC in urine are the most relevant and rather complementary. Notably, both have been recommended by many countries for biological monitoring of occupational exposure to DMF.

Thus, tNMF and AMCC in urine were selected as the BME for deriving HBM-GV_Worker_ for DMF biomonitoring.

#### 3.4.7. Analytical Methods

The analytical protocols and methods for HBM measurement of DMF in HBM4EU participants’ member states are detailed in the “Prioritised list of biomarkers, matrices and analytical methods for the 2nd prioritization round of substances” [87]. NMF is usually measured in urine samples of exposed workers without prior derivatization using GC-MS (gas chromatography-mass spectrometry), GC-NPD (Nitrogen-phosphorus detectors), or GC-FPD (Flame Photometric Detector). Limits of detection are between 0.2 (GC-NPD) and 0.5 mg·L^−1^ (GC-MS). Kawai et al. (1992) reported that results can be affected by the temperature of the GC injection port [32]. Therefore, a temperature of 250 °C or above is recommended to obtain complete degradation of HMMF into NMF to measure tNMF in urine. AMCC in urine samples has been analyzed by a combination of liquid chromatography with mass spectrometry and LC-MS/MS after solid phase extraction. LODs of 5 and 5.5 μg L^−1^ were achieved [82,88].

### 3.5. Published Limit Values for Urine tNMF and AMCC in Occupational Setting

Recognized national and international agencies or organizations provide limit values for tNMF and/or AMCC in urine. Table 4 compiles these proposed limit values, indicating how they were produced.

As shown in Table 4, available data allowed the derivation of limit values for tNMF and AMCC in urine via two approaches. These approaches correspond to the first and second options described in the HBM4EU project (Figure 1). Option 1 was retained by ACGIH experts who derived limit values for both tNMF and AMCC in urine from workplace studies showing relationships between concentrations of these biomarkers and health effects. Option 2 was used by SCOEL and DFG, who produced limit values for tNMF (SCOEL and DFG) and AMCC (DFG) using correlations between external exposure and the concentration of biomarkers and based on the occupational exposure limit (OEL) for DMF. As shown in Figure 1, option 2 should be used for the determination of HBM-GVs only in those cases where option 1 is inapplicable. Moreover, concerning DMF, option 2 is not the best choice, as this substance is readily absorbed through the skin. This is abundantly documented by experimental data and in the workplace [27,47,80]. The absorption of DMF vapor through the skin and by inhalation was evaluated in a study from Nomiyama et al. (2001) [26]. In this study, it was estimated that skin and lung absorption contributed to 40% and 60% of vapor absorption, respectively. DMF absorption through the skin is even higher when direct contact with liquid DMF is possible (see Section 3.1). For these reasons, the limit values for tNMF and AMCC proposed by SCOEL and DFG are not the first choice. Thus, option 1 is preferred for deriving an HBM-GV (Figure 1). This is the option which was retained by ACGIH experts [16]. Their proposal of a 30 mg·L^−1^ (end of shift) limit value was based on the results of the studies by Lauwerys et al. (1980), Sakai et al. (1995), Fiorito et al. (1997), Wrbitzky (1999), and He et al. (2010) [27,45,48,50,55]. It did not take into account the more recent publications by Kilo et al. (2016) and Wu et al. (2017) [46,51]. In the same way, the ACGIH proposal of a 30 mg·L^−1^ limit value for AMCC in urine was based on only two publications [45,55], and did not take into account the more recent studies by Kilo et al. (2016) and Wu et al. (2017) [46,51].

Finally, none of the limit values previously published for urine tNMF and AMCC in occupational settings can be retained, either because of the inadequacy of the method applied for their production (SCOEL, DFG) or because several essential publications were not taken into account for their elaboration (ACGIH). Consequently, the identification of a POD for the production of HBM-GV_Worker_ for tNMF and AMCC in urine must be performed, using the option 1 method of the HBM4EU decision tree (Figure 1).

### 3.6. Choice of Key Studies and Identification of a POD for tNMF in Urine

Four of the occupational studies reporting the association of urine tNMF concentrations with hepatic damage or its absence (Table 1) cannot be retained because of methodological flaws:

- the study by Lyle et al. [52], because it reports imprecise results with evident errors in measurements and/or units of t-NMF concentrations in urine;
- the study by Yonemoto et al. [53], because the unit used for tNMF urinary excretion (mg·d^−1^) is inadequate for deriving HBM-GV_Worker_; and
- the papers by Catenacci et al. (1984) [54] and Fiorito et al. (1997) [50], because the analytic method used by these authors for tNMF measurements gave underestimated results [16,18,20].

Considering the other six studies testing for hepatic damage associated with tNMF concentration in urine, three studies were conducted in European countries and the other three in Asian countries.

Despite the interest and relevance of the studies conducted by Lauwerys et al. (1980) [27], Wrbitzky and Angerer (1998) [47], and Wrbitzky (1999) [48], they cannot be retained to derive an HBM-GV_Worker_ because of the flaws detailed below:

- Lauwerys et al. (1980) [27] observed no effect on liver enzymes up to 40–50 mg·g^−1^ creatinine (cr) tNMF in urine of 22 workers exposed to DMF during five consecutive days. The authors underlined that in the factory, the selection criteria (not disclosed) at the beginning of employment were rather severe and could have led to recruitment bias so that the results obtained may not reflect responses in any worker [16];
- The two publications by Wrbitzky and Angerer (1998) and Wrbitzky (1999) reporting on the same study conducted in a cohort of 126 workers showed that liver damage was significantly more frequent in the exposed group than in controls. Mean tNMF concentration in the exposed group was 9.1 mg·g^−1^ creatinine (14.9 mg·L^−1^). However, considering the working areas, it was observed that liver damage was unexpectedly associated with the lowest exposure group (mean urine tNMF: 4.5 mg·g^−1^ creatinine) and could be explained by a higher alcohol consumption. In the other three areas, no excess of liver damage was observed for mean urine tNMF concentrations of 6.7, 11.6, and 16 mg·g^−1^ creatinine [47,48].

Finally, four studies can be selected to derive an HBM-GV_Worker_:

- In a recent European study by Kilo et al. (2017), no excess risk of liver damage was observed in a cohort of 220 workers exposed to DMF with a mean concentration of 7.75 mg·L^−1^ tNMF in urine, compared with 175 controls [51].
- The other three studies were conducted in Asian people:
- Sakai et al. (1995) reported no effects on liver enzymes of DMF exposure in 10 workers during à 2.5-year-follow-up. The mean tNMF concentration in the urine of these workers was 24.47 mg·g^−1^·cr [55];
- He et al. (2010) also reported that, when their cohort of 79 workers was divided into three groups, a significantly elevated risk of liver damage (liver enzyme elevation) according to DMF exposure was observed only in the group with the highest exposure (mean tNMF concentration: 26.5 mg·g^−1^·cr) [45]; and
- Wu et al. (2017) measured liver enzyme activity in a cohort of 698 workers exposed to DMF and in 188 controls. They also measured tNMF urine concentration in exposed workers. A significantly elevated risk of liver damage was observed only for the third tertile of tNMF distribution (median tNMF concentration: 9.59 mg·L^−1^). The lower limit for the benchmark dose with a benchmark response of 10% above the adverse response rate of liver injury seen in the control group (BMDL_10_) was 14 mg·L^−1^ (tNMF) [46].

From the above data, it appears that liver damage was observed in groups of workers with mean tNMF concentrations in urine of:

- 26.5 mg·g^−1^·cr [45] or
- a median concentration of 9.59 mg·L^−1^ and a BMDL_10_ value of 14 mg·L^−1^ [46].

No liver damage was observed when the mean tNMF concentration in urine was:

- 7.75 mg·L^−1^ [51] or
- 9.6 mg·g^−1^·cr [45] or
- 24.47 mg·g^−1^·cr [55].

Based on the overall results, it seems appropriate to identify a value of 10 mg·L^−1^ or 10 mg·g^−1^ cr as an HBM-GV_Worker_ for tNMF in urine to prevent health effects of DMF exposure.

### 3.7. Choice of the Key Study and POD for AMCC in Urine

As described above, the biomonitoring of the urinary AMCC is relevant to evaluate DMF cumulative exposure of the previous days. Moreover, AMCC results from the formation of MIC, the reactive intermediate probably responsible for DMF hepatotoxicity. There are few studies providing data on the association of urinary AMCC concentrations with liver damage. However, the same four studies which were used for the elaboration of an HBM-GV_Worker_ for tNMF can be used to derive an HBM-GV_Worker_ for AMCC [45,46,51,55]:

- In the German study by Kilo et al. (2016), no effects on liver enzymes were observed in a cohort of 220 workers with a mean AMCC urine concentration of 9.42 mg·g^−1^ cr when they were compared to 175 controls; however, the range of the measured AMCC urine concentrations was very large (standard deviation: 10.42 mg·g^−1^ cr) [51];
- in the Japanese study by Sakaï et al. (1995), no effects on liver enzymes were observed in 10 workers exposed to DMF during à 2.5-year follow-up. The mean AMCC concentration in the urine of these workers was 22 mg·g^−1^·cr [55];
- in the study from China, He et al. (2010) also reported that, when their cohort of 79 workers was divided into three groups, a significantly elevated risk of liver damage (liver enzyme elevation) according to DMF exposure was observed only in the group with the highest exposure (mean AMCC concentration in urine: 45.5 mg·g^−1^ cr). Geometric mean values for the concentration of AMCC in urine of workers from the low and medium exposure groups were 4.25 mg·g^−1^·cr and 25.4 mg·g^−1^·cr, respectively [45];
- a second Chinese study by Wu et al. (2017) measured liver enzyme activity in a cohort of 698 workers exposed to DMF and in 188 controls. They also measured AMCC urine concentration in exposed workers. A significantly elevated risk of liver damage was observed only for the second and the third tertiles of the AMCC distribution (median AMCC concentrations: 44.09 mg·L^−1^ and 148.01 mg·L^−1^, respectively). The median and maximal AMCC concentrations in the low exposure group (1st tertile), with no detectable liver damage excess, were 2.18 mg·L^−1^ and 16.95 mg·L^−1^, respectively. The lower limit for the benchmark dose with a benchmark response of 10% above the adverse response rate of liver injury seen in the control group (BMDL_10_) was 155 mg·L^−1^ (AMCC) [46].

Based on these four studies, NOAEL values for AMCC in urine are between 9.42 mg·g^−1^·cr and 25.4 mg·g^−1^·cr with LOAEL of 44 mg·L^−1^ or 45.5 mg·g^−1^·cr. Due to the large interval of NOAEL values and the large margin between NOAEL and LOAEL values, an HBM-GV_Worker_ of 10 mg·g^−1^·cr is recommended for AMCC in urine. This proposal is conservative.

## 4. Discussion

Literature on DMF biomonitoring offers a large database on robust relationships between health effects and urine levels of tNMF, and to a lesser extent of AMCC, the critical effect being hepatic damage (characterized by an elevation of serum hepatic enzyme activity). Limit values have previously been proposed by SCOEL (2006), ACGIH (2017), and DFG (2019) for tNMF and AMCC in urine of workers exposed to DMF [16,19,20]. However, a recent study by Wu et al. (2017) identified a lower threshold value for hepatotoxic effects than those previously identified by SCOEL, ACGIH, or DFG for tNMF concentration in urine (15 mg·L^−1^ to 30 mg·L^−1^: see Table 4) [46].

Moreover, in its opinion on a restriction dossier for DMF [7], the RAC preferred deriving the DNEL_inhalation_ value for workers from the Kilo et al. (2016) study [51], which was also not taken into account for the evaluations by SCOEL (2006) [20] and ACGIH (2017) [16].

This background justified a new evaluation of the published data for the derivation of guidance values for tNMF and AMCC concentrations in urine of workers.

The present evaluation identified a pool of studies for the derivation of an HBM-GV_Worker_ for tNMF in urine. From the results provided by these studies, an HBM-GV_Worker_ of 10 mg·L^−1^ (or 10 mg·g^−1^·cr) was agreed on and is hereby recommended. Considering tNMF half-life time, end-of-shift urine sampling at any day of the workweek can be performed for this biomarker.

In addition to this recommendation, an HBM-GV_Worker_ has been derived for AMCC which is an indicator of cumulative exposure after several days. Due to the dispersion of NOAEL values and the large margin between NOAEL and LOAEL values, a conservative limit value of 10 mg·g^−1^·cr was agreed on and is provisionally recommended. Considering AMCC half-life time, end-of-shift urine sampling at any day of the workweek can be performed for this biomarker.

In the current state of knowledge, tNMF should be considered as the most reliable biomonitoring indicator for DMF exposure. As tNMF and AMCC are not alternative, but rather complementary biomarkers of DMF exposure, further studies on the association of AMCC urine level with health effects in workers exposed to DMF should be encouraged. They will probably allow the identification of a higher HBM-GV_Worker_ for AMCC.

Acute and chronic alcohol consumption interferes with DMF metabolism and tNMF and AMCC elimination kinetics; thus, information on alcohol habits and alcohol consumption on the sampling day should be collected to allow interpretation of the measurement results.

As DMF LOAEL and NOAEL for developmental effects in the most sensitive species are higher than the corresponding values for hepatotoxic effect, and as DMF carcinogenic effects in animals follow hepatic damage, the HBM-GV_Worker_ for tNMF and AMCC which were established to protect against hepatotoxic effects are expected to also protect from developmental toxicity and carcinogenic effects. Alcohol intolerance can be observed at lower exposure levels in some individuals. Consequently, workers exposed to DMF should be informed of the risk of alcohol consumption in the exposure periods and at least a week after their end.

As mentioned in Apel et al. (2020), a level of confidence (low, medium, or high) could be attributed to each calculated HBM-GV. The level of confidence should reflect the uncertainties identified during the derivation of the value and could constitute a good incentive to later revise values with an estimated ‘lower’ level of confidence [2].

An attribution of a global level of confidence is suggested for the proposed HBM-GV_Worker_ (for both BME), considering the assessment of the various uncertainties, and detailed in the table below (Table 5).

It should be specified that the levels of confidence proposed in this document are not determined according to an established recognized methodology, but rather rely on expert judgment regarding the reliability of the data and the calculation method used to derive the HBM-GVs.

## 5. Conclusions

The present work provides an HBM-GV_Worker_ which can be used to assess and limit occupational DMF exposure. In the current state of knowledge, tNMF should be considered as the most reliable biomonitoring indicator for DMF exposure. As tNMF and AMCC are not alternative, but rather complementary biomarkers of DMF exposure, further studies on the association of AMCC urine levels with health effects in workers exposed to DMF should be encouraged. They will probably allow the derivation of a higher HBM-GV_Worker_ for AMCC.

Moreover, it is important to underline that a population living near industries using/producing DMF may also be significantly exposed [93]. Studies for the characterization of DMF environmental exposure and its effects should be encouraged before considering the elaboration of an HBM-GV_GenPop_.

## Figures and Tables

**Table 2:** Lower points of departure for relevant adverse effects reported in animal studies.

Route	Effects on Liver	Reproductive Effects	Carcinogenic Effects on Liver
Inhalation	NOAEL: 25 ppm LOAEL: 100 ppm (Rats and mice) [68]	NOAEL: 25 ppm LOAEC: 150 ppm (Rabbits) [76]	LOAEC: 200 ppm (mice) LOAEC: 400 ppm (rats) [67]
Oral	NOAEL = 238 mg/kg bw/d LOAEL = 475 mg/kg bw/d (Rats) (BASF (1977) unpublished data, quoted by ECHA [7])	NOAEL = 166 mg/kg bw/d LOAEL = 503 mg/kg bw/d (Rats) [76] NOAEL: 44.1 mg/kg bw/d (Rabbits) [78]	LOAEL = 800 ppm (Rats) [69]
Dermal	-	LOAEL: 94 mg/kg/d (Rats) 100 mg/kg/d (Rabbits) [76]	-

**Table 3:** Advantages and limits of the relevant BME.

Analyte	Biological Matrix	Advantages	Limits
Total NMF	Urine	- Short half-life: concentration at the end of shift is a good estimate of the exposure of the same day - Good specificity: not found in the general population - Strong association with health (hepatic) effects - Good correlation with airborne DMF	- Delayed excretion after skin absorption - Influenced by alcohol consumption - Analytical methods should be adapted to the measure of tNMF (NMF + HMMF)
AMCC	Urine	- Long half-life: concentration at the end of shift and at the end of the week is a good estimate of the exposure during the workweek - Elimination not delayed by skin exposure - Directly linked to MIC formation and hepatotoxic effects - Good correlation with mean airborne DMF concentration of the preceding days	- Might be found in general population, especially in active or passive smokers
MCVal	Blood	- Very stable, good indicator of the cumulated exposure of the last months - Directly linked to MIC formation - Dose response association with health (hepatic) effects - Acceptable correlation with airborne DMF	- Limited data on the associations with external exposure and health effects - Invasive sampling - High technical requirements and cost of the measurement
DMF	Urine	- Specific	- Limited data on the associations with external exposure and health effects - Very short half-life (2 h) - Only low levels excreted at high absorbed doses.
Formamide	Urine	None	- No data on the associations with external exposure and health effects - Not specific, can be found in the absence of DMF exposure.

**Table 4:** Existing limit values for urine tNMF and AMCC in an occupational setting.

Agency	Reference Value for Airborne DMF (Key Studies and Critical Effect)	Biomarker	Approach/ Endpoint	Key Study	Internal TRV and Sampling Time
SCOEL, 2006 [20]	8h-TWA = 5 ppm (Liver damage in rats and mice, exposed by inhalation, whole body) [68]	tNMF in urine	Correlation based on the OEL of 5 ppm	Studies in workers [32,48,55,79,80,81,89,90]	BLV = 15 mg·L^−1^ Post-shift
ACGIH, 2017 [16]	TLV-TWA = 5 ppm Liver damage in rats and mice and irritation in humans (eyes and upper respiratory tract) [49,68,91,92]	tNMF in urine	Relation between BME levels and effects on liver	Studies in workers [27,45,48,55]	BEI = 30 mg·L^−1^ End of shift
		AMCC in urine	Relation between BME levels and effects on liver	Studies in workers [45,55]	BEI = 30 mg·L^−1^ End of shift and end of workweek
DFG, 2019 [19]	MAK value = 5 ppm (Liver damage in rats and mice, exposed by inhalation, whole body) [68]	tNMF in urine	Correlation based on the MAK value of 5 ppm	Studies in workers [82]	BAT = 20 mg·L^−1^ End of exposure or end of shift
		AMCC in urine			BAT = 25 mg·g^−1^·cr End of exposure or end of shift; Long-term exposure indicator: sampling at the end of a shift after several previous shifts

BLV: Biological limit value; BEI: Biological Exposure Index; BAT: Biologischer Arbeitsstoff Toleranzwert; 8h-TWA: Time weighted average on 8 h; TLV-TWA: threshold limit value—time weighted average; MAK value: Maximale Arbeitsplatz-Konzentration.

**Table 5:** Level of Confidence (LoC) regarding HBM-GV_Worker_ recommended for urinary tNMF and AMCC.

	Urinary tNMF	Urinary AMCC
Regarding the nature and quality of the toxicological data	The database on DMF is based on a large number of both human and animal studies, and data on tNMF are robust and consistent. LoC: High	The database on DMF is based on a large number of both human and animal studies, but available studies reporting results for AMCC are limited. LoC: Medium
Regarding the critical endpoint and mode of action	The confidence in the evidence of effects on the liver function is high. The effects on the liver after DMF exposure are well-studied in humans (workplace) and animals. LoC: High	The confidence in the evidence of effects on the liver function is high. The effects on the liver after DMF exposure are well-studied (in humans and animals). LoC: High
Regarding the selected key studies for identification of the POD and their results	The database gives several robust occupational studies with many subjects and consistent results for tNMF. The approach consists of the selection of a pool of studies (from 1980 to 2017) carried out on Asians and Caucasian people (to consider the genetic variability due to ethnicity). LoC: High	The HBM-GV_Worker_ is based on a the same four studies used for the derivation of tNMF HBM-GV_Worker_. However, the results of these studies indicate a large interval of NOAEL values together with a large margin between NOAEL and LOAEL values. LoC: Low
Global LoC	High	Low-medium

## Data Availability

Not applicable.
